# Chronic Endometritis: Old Problem, Novel Insights and Future Challenges

**DOI:** 10.22074/ijfs.2020.5779

**Published:** 2019-11-11

**Authors:** Elena Puente, Luis Alonso, Antonio Simone Laganà, Fabio Ghezzi, Jvan Casarin, Jose Carugno

**Affiliations:** 1 Assisted Reproduction Unit, Fertia Clinic, Fuengirola, Málaga, Spain; 2 Unidad de Endoscopia Ginecológica, Centro Gutenberg, Málaga, Spain; 3 Department of Obstetrics and Gynecology, "Filippo Del Ponte" Hospital, University of Insubria, Varese, Italy; 4 Obstetrics and Gynecology Department, University of Miami, Miller School of Medicine, Miami, Florida, USA

**Keywords:** Endometritis, Hysteroscopy, Immunochemistry, Reproductive Outcomes

## Abstract

Chronic endometritis (CE) is a poorly investigated pathology which has been related to adverse reproductive out- comes, such as implantation failure and recurrent miscarriage. In this paper, we aim to provide an overview of diag- nosis, etiology, pathophysiology and treatment of CE, its impact on endometrial microenvironment and its associa- tion with infertility. We present a narrative review of the current literatures, synthesizing the findings retrieved from searches of computerized databases. CE is more prevalent in infertile patients. Effective antibiotic treatment of CE seems to improve the pregnancy and live birth rate in patients with unexplained recurrent pregnancy loss (RPL), and increase ongoing pregnancy rate in patients with recurrent implantation failure. In order to increase the diagnostic ac- curacy, immunohistochemistry is recommended besides the conventional histology. In addition, hysteroscopy could be considered as gold standard tool for diagnosis, considering its high correlation with histological findings. CE, as the chronic inflammation of endometrium, is usually asymptomatic and probably underestimated. Interaction of bac- teria with endometrial microenvironment promotes changes in leukocyte population, cytokine production and growth factors which support its negative impact on endometrial receptivity. Nevertheless, standardization of the criteria for histopathological diagnosis and immunohistochemistry technique needs to be defined.

## Introduction

Assisted reproductive techniques undergo great advances, including improved means of tissue culture, updated criteria of embryonic selection and extended culture to blastocyst, leading to reach pregnancy rates up to 66% in the selected patients (1-4). In this regards, many factors involved in the implantation are not yet fully understood and it seems that endometrium plays much more relevant role than the other recognized factors (5-7).

Endometrium is a unique tissue that undergoes monthly cyclical changes resulting in menstruation, proliferation, secretion and decidualization under the influence of ovarian steroids. Endometrium contains a large variety of immunocompetent cells, natural killers (NKs), macrophages, T cells and neutrophils, whose composition and density fluctuates periodically (8). The cycle-dependent changes in these subpopulations of leukocytes and their mediators probably play a crucial role in implantation. In contrast, antibody-bearing B-lymphocytes and plasma cells are rarely found in endometrial tissue (9).

Chronic endometritis (CE) is defined as localized inflammation of the endometrial mucosa characterized by the presence of edema, increased stromal cell density, dissociated maturation between epithelial cells and stroma fibroblasts, as well as the presence of plasma cell infiltrate in the stroma (10). These changes at the level of endometrial microenvironment could affect endometrial receptivity (11).

CE is often asymptomatic or present with non-specific clinical symptoms, such as pelvic pain, dysfunctional uterine bleeding, dyspareunia, vaginal discharge, vaginitis, recurrent cystitis and mild gastro-intestinal discomfort (12). Nonspecific quality of the symptoms and importance of performing endometrial biopsy to confirm diagnosis makes it difficult to estimate the prevalence of this condition.

Based on endometrial biopsy of patients who subsequently underwent hysterectomy with benign pathology, prevalence of CE is 10-11% of the general population (13, 14), 3-10% of patients with abnormal uterine bleeding (15) and up to 72% of women with suspected pelvic inflammatory disease (PID) due to the sexually transmitted diseases (STDs) (16). As far as infertile patients are concerned, the prevalence varies greatly depending on the utilized biopsy method and investigated population. In a prospective study published by Cicinelli et al. (17) in a total number of 2190 diagnostic hysteroscopy with different indications, they found a prevalence of 20% (438 patients) with CE, among whom 37% were also infertile. However, Kasius et al. (18) reported a prevalence of only 2.8%, in a total of 678 women.

CE can be due to the presence of foreign bodies or structural pathology of the endometrial cavity, such as the presence of intrauterine device (IUD), submucous myomas, polyps, retained products of conception, incomplete abortion or infectious agents. The most frequent infectious agents are common bacteria frequently found in the urogenital area such as Streptococcus (27%), E. coli (11%), Enterococcus faecalis (14%) and Ureaplasma urealyticum (11%) (19). The presence of Chlamydia trachomatis is only 2.7%, and Neisseria gonorrhoeae is practically undetectable as causative in CE (20). These findings are in line with the results of the PEACH study (21), showing that 60% of women with PID present non-gonococcal or Chlamydia infection.

In certain areas of the world, Mycobacterium tuberculosis is highly prevalent. It is considered as the main cause of infertility in 40-75% of cases, since it causes implantation failure due to alteration of the immune response at the endometrial level, hormonal alterations and release of antiphospholipid antibodies (22). Today, it is well accepted that the uterus is not a sterile cavity, and that presence of the microorganisms is not equal to inflammation (23).

Asymptomatic presence of bacteria in the endometrial cavity in either transcervical samples, or cultures obtained in post-hysterectomy specimens, has been reported by several investigators (24, 25). More recently metagenomics, investigating hypervariable regions of the ribosomal 16S rRNA genes allow definition of genus order and species of bacteria, leading to confirm presence of up to 12 different bacterial types in up to 95% of endometrial biopsies performed in patients undergoing hysterectomy for non-cancer indications (26). As described by Espinoza et al. (27), accumulating evidences suggest that endometrium is continuously exposed to bacteria from the genital tract. Therefore, presence of pathology is also determined by interaction of the infectious agent with the endometrial microenvironment (28).

Considering that the published data has not yet been able to draw a firm conclusion in this regard, in this paper we aim to review the current pieces of evidence regarding diagnosis, impact on reproductive outcomes and management of CE.

### Pathophysiology of the endometrial microenvironment, microbial and immune cross-talk

In the normal endometrium, B lymphocytes are only located at the basal layer, representing less than 1% of the leukocyte population. Conversely, in CE a large population of B cells lymphocytes are present at the both basal layer of the endometrium and glandular epithelium, as well as in the lumen of endometrial glands (29). Recent data suggest that a lipopolysaccharide derived from E. coli is capable of inducing the in vitro expression of E-selectin, as an adhesin that promotes passage of B cells to the endothelium of endometrial microvascularization (30). In addition, E-selectin promotes expression of chemoattractant CXCL13, activating adhesion molecules of B cells and expression of CXCL1 at the glandular endometrium level (8). In this microenvironment, gram-negative bacteria within the endometrium induce an abnormal immune response with migration of circulating B lymphocytes to the endometrial stromal compartment (8). At the endometrial level, plasma cells of the stroma express multiple immunoglobulins (IgM, IgA1, IgA1, IgG1 and IgG2), while excess of these antibodies could negatively affect implantation of the embryo (31).

In a study performed by Di Pietro et al. (32), expression of the 25 genes encoding the proteins involved in inflammation, proliferation and apoptosis at endometrial was compared by real-time polymerase chain reaction (RT-PCR) during the implantation time window in 16 women with hysteroscopic and histological diagnosis of CE and 10 healthy women without endometritis; the results of this study suggested that endometrial expression of some genes is significantly altered. In particular, they found up-regulated gene expression of insulin-like growth factor binding protein 1 (IGFBP1), B-cell CLL/ Lymphoma 2 (BCL2) and BCL2-associated X protein (BAX), while down-regulated gene expression of IL11, Chemokine (C-C MOTIF) Ligand 4 (CCL4), insulin-like growth factor 1 (IGF1) and caspase 8 (CASP8). These altered gene expressions could affect, at least in part, the embryonic implantation and they also explained presence of endometrial hyperplastic lesions.

In CE, stromal cells secrete IGFBP1 protein during the decidualization process, exerting a negative effect on the implantation process and counteracting effect of IGF2. Thus, an increase of IGFBP1 expression and reduction of IGF1 expression in CE may lead to unfavorable conditions for implantation and embryonic development (33).

In this scenario, CE can alter the production of cytokines, impair endometrial function and induce an abnormal pattern of the leukocyte population at the endometrial level, leading to altered secretion of paracrine factors involved in endometrial receptivity. As reported elsewhere, decrease in IL11 production by epithelial and stromal cells may lead to dysregulation of trophoblastic invasion, associating with infertility. Similarly, lower CCL4 activity in CE may lead to a decreased recruitment of NKs and macrophages, accounting for the observed implantation failure (34). In addition, downregulation of BCL2 and CASP8 (35), associated with upregulation of BAX (36), causes endometrial cell resistance to apoptosis and disturb the correct process of implantation (35). 

### Diagnosis of chronic endometritis: current management and potential pitfalls

Diagnosis of CE represents a challenge for the gynecologist. The clinical manifestations of CE such as pelvic pain, vaginal discharge, dyspareunia and abnormal vaginal bleeding are non-specific, while about 25% of patients with CE are asymptomatic (37). Moreover, the peripheral blood inflammation markers, such as C-reactive protein (CRP), leukocytosis, leptin and IL6 do not predict presence of CE (8). 

### Histopathology of chronic endometritis

**Fig 1:**
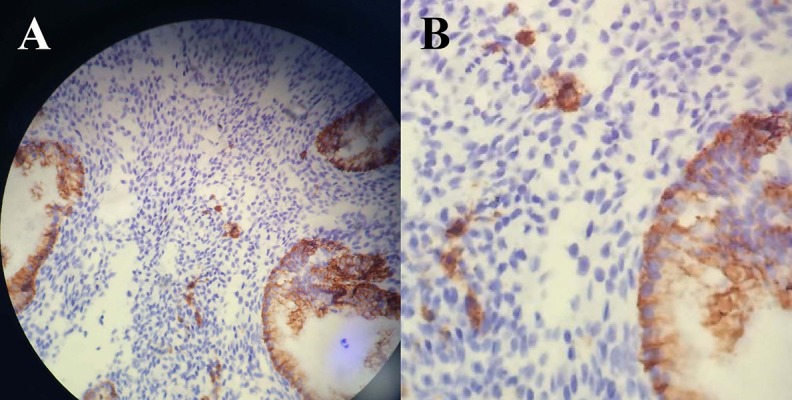
: Immunochemistry of chronic endometritis. **A.** Fragment of endometrial biopsy specimen showing glandular cell surface syndecan 1 immunoreactivity. Plasma cells are highlighted by syndecan 1 staining in the center of the picture (original magnification: ×400) and **B.** More detailed picture of plasma cell syndecan 1 immunoreactivity.

The accepted gold standard for diagnosis of CE is presence of the plasma cells in endometrial tissue. However, their histological identification is sometimes hampered by the presence of mononuclear cell infiltration, mitosis and proliferation of stromal cells, plasmacytoid appearance of stromal cells (fibroblasts and mononuclear cells) or decidual transformation of the endometrium during late secretory phase. Plasma cells are characterized by the presence of chromatin in the form of a clock face inside an eccentric nucleus with perinuclear halo (Fig .1) (15).

Syndecan 1 is a proteoglycan of the transmembrane heparan sulfate type presenting on the surface of plasma cells and keratinocytes, while it is not expressed in mononuclear cells, lymphocytes or endometrial stromal cells. It is also known as CD138, facilitating detection of plasma cells and presence of CE, not affected by intra- and inter-observer variability (38).

It is recommended to include clinical immunohistochemistry and conventional pathology study to increase the accuracy of the CE diagnosis (15). Furthermore, it is important to obtain standardization of the current diagnostic techniques, considering that depending on the dilution of Syndecan 1, diagnosis of CE might differ. For a dilution of 1:1000, a prevalence of 2.8% was initially reported for CE in asymptomatic infertile women prior to *in vitro* fertilization (IVF) (39), which does not overlap with the prevalence of 30.3%, previously reported by Johnston-MacAnanny et al. (40), as well as the prevalence of 10% in the patients with recurrent miscarriage (41). In addition, the menstrual cycle phase whereby the biopsy is performed and thickness of the biopsy have paramount importance: in particular, in 15% of the samples during secretory phase, plasma cells are present only in the basal layer of the stroma, which will be missed if not included in the biopsy. Finally, it is important to define number of the plasma cells required to establish diagnosis of CE: although most authors believe that there must be two or more plasma cells, the others recommend presence of five or more plasma cells in at least one of the three sections of biopsy (40).

### Hysteroscopic findings of chronic endometritis

**Fig 2:**
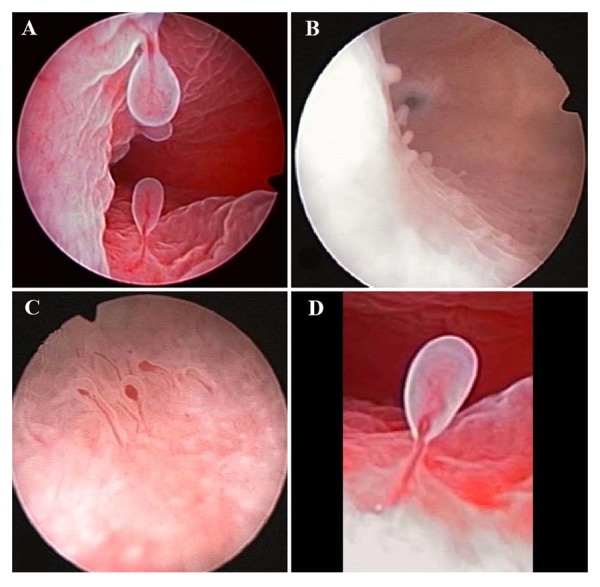
Different findings of chronic endometritis at the fluid hysteroscopy. **A.** Endometrial surface is completely covered by micropolyps, **B.** Isolated micropolys on the lateral wall of the cavity, **C.** Endometrial mucosa appears thick, edematous, diffuse hyperemic, with presence of micropolyps, and **D.** Detailed image of an endometrial micropolyp appearance.

Hysteroscopy is a useful diagnostic modality in CE. Usual hysteroscopic findings for characteristic CE include presence of local or diffuse hyperemia, edema of the stroma and presence of micropolyps (less than 1 mm in size, Fig .2) (42).

Cicinelli et al. (42, 43) reported that presence of endometrial micropolyps at hysteroscopy suggests the existence of CE. Interestingly, they obtained a positive diagnostic correlation of 93.4% with the pathology findings, following their criteria of hysteroscopic diagnosis. These findings have been replicated by others (44) with 86.5% correlation of hysteroscopic with histological diagnosis.

### Chronic endometritis and reproductive outcomes

The implantation consists of a physiological process involving mediators of inflammation such as leukocytes, cytokines, chemokines and other endometrial factors. All these cells and their mediators play an essential role in the regulation of immunoresponse and growth of the trophoblast. The presence of CE can alter receptivity of the endometrium creating an inadequate microenvironment that interferes with normal implantation. In particular, recent data (8) suggests that the endometrium of one third of infertile patients, presenting with CE, expresses high level of estrogen receptor, progesterone and Ki-67 nuclear marker of cell proliferation in both epithelial cells and stroma, in addition to the increased expression of anti-apoptosis genes such as BCL2 and BAX, all of which represent a proliferative phenotypic change of the endometrium even in the secretory phase. This increase in expression levels of estrogen and progesterone receptors was replicated by Wu et al. (33), suggesting that CE modifies stromal cells by altering the function of these hormonal receptors. 

CE also modifies the pattern of uterine contractility in both of the periovulatory and mid-luteal phases of menstrual cycle (45). Physiologically, in the proliferative phase, there is anterograde contractility from the fundus to the cervix which facilitates removal of menstrual debris, followed by periovulatory and the luteal phase when there is predominance of retrograde contraction in the opposite direction, from the cervix to fundus, which favors migration of the spermatozoa to the fallopian tubes. Conversely, during CE, there is 3.3 times lower occurrence of retrograde contractility of the fallopian tubes (46). This “altered peristalsis” induced by the presence of CE could impair, at least in part, fertility and contribute to some of the symptoms such as pelvic pain and dysmenorrhea.

### Implantation failure after *in vitro* fertilization and chronic endometritis

Impact of the CE presence in implantation is controversial, although many studies suggest a negative impact on the endometrial receptivity of plasma cells as well as IgM, IgG and IgA alterations in genes encoding proteins involved in the inflammatory response, proliferation and apoptosis.

Bouet et al. (47) reported a prospective observational study including 46 women with recurrent implantation failure (RIF), defined as failure to achieve pregnancy after transferring three good quality embryos in fresh or frozen cycle in women up to 35 years of age, or 4 embryos of good quality in women over 35 years. In this study, the authors excluded women with uterine cavity anomalies, presence of submucous myomas or endometrial polyp of more than 5 mm, as well as the patient who were treated with antibiotics within one month prior to biopsy or those who had unexplained vaginal bleeding. Hysteroscopy with endometrial biopsy was performed between days 6 and 12 of the cycle. Diagnosis of CE was confirmed by 1:100 immunohistochemical dilution, while they were considered positive with the presence of 5 or more plasma cells in 10 high power fields (×400). They found 14% prevalence of CE, with 80% correlation between hysteroscopic criteria and histological confirmation. Using a slightly different methodology, others (40) investigated retrospectively 33 women, defining implantation failure as the failure to achieve pregnancy after two cycles of IVF with transfer of at least one good-quality embryo. They performed endometrial biopsy and immunohistochemical study dilution of 1:100, considering it negative with the presence of less than one plasma cell, reporting a prevalence of 30.3% CE. In a larger cohort analysis, Cicinelli et al. (48) included the patients who were younger than 40 years, normal responders at ovarian stimulation and normal karyotype, defining RIF after embryo transfer of at least six good quality embryos in three or more previous IVF/intracytoplasmic sperm injection (IVF/ICSI) cycles. Patients with follicle stimulating hormone (FSH)>10 on day 3, body mass index (BMI)>30, endometriosis, history of abortion, steroid use, autoimmune disease, antiphospholipid syndrome, thrombophilia or presence of anti-spermatozoid antibodies were excluded from the study. They performed hysteroscopy and biopsy in the follicular phase of the following cycle, obtaining an endometrial biopsy and cultured these cells. According to their data analysis, CE was diagnosed by hysteroscopy in 66% of the cases, by histology in 57.5%, and by positive culture in 45% of the cases. Higher rate of positive diagnosis could be explained probably by the experience of pathologist and hysteroscopist, regarding the diagnostic criteria of CE (40) and a selection bias since the authors’ Institution is a referral center for women with suspected CE. The final concordance between hysteroscopic and histologic diagnosis of CE was 87%. Noteworthy to say that women who were included in both studies, performed by Johnston-MacAnanny et al. (40) and Cicinelli et al. (48), were treated with antibiotic. Patients included in the former study (40) were treated with 100 mg Doxycyclin for two weeks, followed by Ciprofloxacin and Metronidazole 500 mg (twice daily) for two weeks in those with positive cultures. Those included in the latter study (48) were treated with Ciprofloxacin 500 mg (twice daily) for 10 days against gram negative bacteria and AmoxicillinClavulanic acid 1 g (twice daily) for 8 days against gram positive bacteria. If the cultures persisted positive, then the antibiotic protocol was repeated up to three times and if the cultures were negative, the patient would receive intramuscular single dose of Ceftriaxone 250 mg, followed by Doxycycline 100 mg (twice daily) and Metronidazole 500 mg (twice daily) for 14 days.

Regarding reproductive outcomes, Cicinelli et al. (48) found a live born rate of 61% in patients responding to antibiotics, whereas the live born rate was only 13% in patients who did not respond to antibiotic therapy. Conversely, in the study performed by Johnston-MacAnanny et al. (40), patients of the CE group improved pregnancy rate after good response to therapy, although the CE group had still lower pregnancy rate than non-CE group, despite a good response to treatment with antibiotics. These different results may probably be due to the other unrecognized endometrial abnormalities, which are not solved with antibiotic therapy.

Overall, both studies suggest that CE has a negative impact on endometrial receptivity, and adequate response to antibiotic therapy may significantly improve reproductive outcomes, as it was confirmed in a recent systematic review and meta-analysis (49). Nevertheless, diagnostic hysteroscopy itself and endometrial biopsy may also play a positive role. In one hand, hysteroscopy could physically remove bacterial biofilms involved in the pathophysiology of CE; on the other hand, endometrial biopsy and the subsequent recovery process can promote secretion of cytokines and growth factors in the endometrium involved in embryo implantation.

### Recurrent pregnancy loss and chronic endometritis

According to European Society of Human Reproduction and Embryology (ESHRE), recurrent pregnancy loss (RPL) is defined as the loss of two or more pregnancy, even not consecutive, occurring before 20 weeks of gestation, which is in agreement with the definition of the American Association of Reproductive Medicine (ASRM) guidelines (50). In patients with implantation failure, the aberrant endometrial microenvironment resulting from an anomalous pattern in the CE lymphocyte population has been linked to RPL. Kitaya et al. (41) reported a total of 58 women with RPL (three or more abortions), detecting presence of CE by immunohistochemistry in 9.3% of the patients. Using the same experiment, others (51) reported a prevalence of 42.9% CE on a total of 142 women with three or more abortions. McQueen et al. (52) studied 395 women with two or more abortions by week 10 or at least one pregnancy loss of more than 10 weeks, finding 9% CE prevalence diagnosed by endometrial biopsy. In the latter study, the patients were then treated with antibiotics: after the first course, there was adequate response in 94% of the cases, rising to 100% after administration of two courses of antibiotics. They reported an increase of live birth rate from 7% before treatment to 56% after receiving antibiotic treatment for two weeks.

Cicinelli et al. (53) performed a retrospective study of 360 women under the age of 40 with three or more abortions before 20 weeks gestation, excluding patients with severe male factor, endometriosis, uterine anomalies, metabolic or hormonal alterations, antiphospholipid syndrome and thrombophilia. Hysteroscopy was performed in the follicular phase. Patients with hysteroscopic diagnosis of CE had endometrial biopsy in the following cycle. They found 57.8% of patient with hysteroscopic sign of CE, out of which 91.3% were confirmed by histology and 68% had positive cell cultures. Confirming previous reports, after antibiotic treatment they found that live birth rate in women responding to antibiotic treatment was higher, compared to non-responder women, suggesting that presence of the infectious agents in the uterine cavity has a potential deleterious impact on the endometrial environment.

Similarly, a more recent case-control observational study (54) was performed in 107 women with two or more abortions before 20 weeks gestation, after ruling out other causes of pregnancy loss. In this study, investigators performed endometrial biopsy analyzed with hematoxylin eosin and CD138, defining CE as the presence of 1-5 plasma cells at immunohistochemistry test. Using these criteria, the prevalence of CE varied from 13% to 56% upon completion of an immunohistochemical study. They also found a trend towards a higher rate of pregnancy loss in women with untreated CE compared to patients without CE. Finally, Bouet et al. (47) published a prospective observational study, including 53 women with two or more unexplained pregnancy loss in pregnancies less than 14 weeks gestation. They performed hysteroscopy and endometrial biopsy, using syndecan 1, as a biomarker, considering that is positive with the presence of five or more plasma cells in 10 high power fields. They found a prevalence of 27% CE. 

## Conclusion

CE is associated with poor reproductive outcomes, including implantation failure and RPL. Accumulating evidences suggest that this condition modifies endometrial microenvironment at different levels: first of all, CE promotes changes on immunocompetent cell population in the endometrium. It also affects production of inflammatory cytokines, involved in NKs recruitment, which play a crucial role in local immune response during early pregnancy and favor implantation. In addition, CE has negative impact on normal endometrial decidualization, promoting proliferation, diminishing apoptosis and modifying the expression of sex steroid receptors, which affect endometrial receptivity. 

Hysteroscopy, in expert hands, could be considered a good tool to combine with histology for diagnosis of CE. Nevertheless, a consensus about strict criteria is mandatory for diagnosis to combine immunohistochemistry with conventional histology. Finally, future investigation should be aimed to redefine the minimum volume of biopsy and the number of plasma cells needed for diagnosis. 

There is still lack of the uniform definition of RPL. Obtaining that would allow more accurate analysis and comparison among different studies. Considering this scenario, part of conflicting data found by different authors can be due to this element.

Antibiotic treatment of CE improves implantation rates and decreases the rate of abortion, although there is a lack of well-designed prospective studies that corroborate this finding.

The metagenomics and a better understanding of the microbioma of the reproductive tract will allow researchers to develop therapies aimed to not only eliminate pathogenic flora but also establish a flora which favors reproductive success.
